# Prioritizing studies of COVID-19 and lessons learned

**DOI:** 10.1017/cts.2021.784

**Published:** 2021-04-21

**Authors:** Dushyantha Jayaweera, Patrick A. Flume, Nora G. Singer, Myron S. Cohen, Anne M. Lachiewicz, Amanda Cameron, Naresh Kumar, Joel Thompson, Alyssa Cabrera, Denise Daudelin, Reza Shaker, Philippe R Bauer

**Affiliations:** 1 University of Miami Miller School of Medicine, Miami, FL, USA; 2 Medical University of South Carolina, Charleston, SC, USA; 3 The MetroHealth System at Case Western Reserve University, Cleveland, OH, USA; 4 The University of North Carolina School of Medicine, Chapel Hill, NC, USA; 5 University of Kentucky, Lexington, KY, USA; 6 Tufts Medical Center, Boston, MA, USA; 7 Medical College of Wisconsin, Milwaukee, WI, USA; 8 Mayo Clinic, Rochester, MN, USA

**Keywords:** Clinical translational research, COVID-19, lessons learned, CTSA, leadership

## Abstract

**Introduction:**

COVID-19 altered research in Clinical and Translational Science Award (CTSA) hubs in an unprecedented manner, leading to adjustments for COVID-19 research.

**Methods:**

CTSA members volunteered to conduct a review on the impact of CTSA network on COVID-19 pandemic with the assistance from NIH survey team in October 2020. The survey questions included the involvement of CTSAs in decision-making concerning the prioritization of COVID-19 studies. Descriptive and statistical analyses were conducted to analyze the survey data.

**Results:**

60 of the 64 CTSAs completed the survey. Most CTSAs lacked preparedness but promptly responded to the pandemic. Early disruption of research triggered, enhanced CTSA engagement, creation of dedicated research areas and triage for prioritization of COVID-19 studies. CTSAs involvement in decision-making were 16.75 times more likely to create dedicated diagnostic laboratories (95% confidence interval [CI] = 2.17–129.39; *P* < 0.01). Likewise, institutions with internal funding were 3.88 times more likely to establish COVID-19 dedicated research (95% CI = 1.12–13.40; *P* < 0.05). CTSAs were instrumental in securing funds and facilitating establishment of laboratory/clinical spaces for COVID-19 research. Workflow was modified to support contracting and IRB review at most institutions with CTSAs. To mitigate chaos generated by competing clinical trials, central feasibility committees were often formed for orderly review/prioritization.

**Conclusions:**

The lessons learned from the COVID-19 pandemic emphasize the pivotal role of CTSAs in prioritizing studies and establishing the necessary research infrastructure, and the importance of prompt and flexible research leadership with decision-making capacity to manage future pandemics.

## Introduction

The COVID-19 pandemic is reminiscent of disease depicted in the fictitious, allegorical, and existential novel by Albert Camus on *La Peste* (the Plague). Taking place in North Africa in the 14th century. Camus describes disease that swept across Europe and North Africa, killing almost 50% of the population. Camus wrote, “They fancied themselves free, and no one will ever be free so long as there are pestilence” [1]. Despite our wealth, technology, and scientific knowledge, the deaths in the current pandemic have been the greatest among the weakest despite their living in the prosperous countries [2].

COVID-19 disease is caused by the novel acute respiratory syndrome coronavirus 2 (SARS-CoV-2). COVID-19 represents an infectious threat of proportions not seen since the 1918 influenza pandemic. Highly contagious, the SARS-CoV2 virus disrupted the economy of the United States (US) and around the world necessitating rapid development of strategies to directly study SARS-CoV-2 with the intent to prevent and/or treat infection and its sequelae effectively and safely. Furthermore, while research centers had been exposed to disasters before, the scale and wide-spread distribution of the disease presented novel challenges. For instance, hurricane Katrina in 2005 raised ethical issues in research, which was not accompanied with adequate policy development in preparation for future crisis at such a large scale [3]. Still the United States came into this crisis with theoretical advantages. Along with tremendous manufacturing capacity, we have an established biomedical research infrastructure. We have extensive expertise in public health, health policy, and basic biology and have previously been able to turn that expertise into new therapies and preventive measures translated into practice. However, shepherding research in the wake of such pandemics or disasters imposed the dual responsibilities on the researchers who may also be the caregivers of protecting the rights of the participants as research subjects, while also ensuring research quality and application of ﬁndings [3]. In addition, the COVID-19 pandemic challenged the calendar and redirected priorities. It was further complicated by science denial [4], and many ignoring advice from established leaders of agencies who are regarded as highly respected and reliable. Many uncertainties regarding COVID-19 disease and its management were present, and the rapid propagation of the pandemic generated immediate challenges.

Research at academic centers was disrupted by the pandemic and many of the challenges faced are addressed in other articles within this issue. However, academic centers also had to adapt to the challenges of studying the virus and COVID-19 disease. A key example is learning how one could safely handle biospecimens from subjects with infection; understanding of the virus’ life cycle, its transmission routes, and risks proved essential to informing biosafety committees. Clinicians had to identify, learn, and adopt best practices for prevention, diagnosis, and management. Registries were created [5] to inform treatment guidelines [6–8]. Gaps in knowledge became rapidly evident [9], revealing opportunities for discovery science including therapeutic studies [9–11]. The flux of ideas was extraordinary and unprecedented regarding speed and quantity and these needed triage and coordination. As there was no central guidance, institutions had to rapidly organize and develop their own approaches to coordinate research; an example occurred in New York, where four distinct hospitals developed a wide variety of strategies to respond rapidly to the COVID-19 pandemic [12].

The Clinical and Translational Science Awards (CTSA) is a program developed by the National Center for Advancing Translational Sciences (NCATS) to emulate innovative solutions that will improve the efficiency, quality, and impact of the process for turning research findings into clinical practice (bench to bedside and back) and improve the health of individuals and the public [13].

The objective of this study was to appraise the impact of the CTSA network’s programs across the country in COVID-19-related research, specifically how CTSA programs adapted to prioritize studies of COVID-19 and lessons learned.

## Methods

NCATS CTSA program conducted a survey of the CTSA network (64 hubs) in October 2020. Center for Leading Innovation and Collaboration (CLIC) from the CTSA/NIH was responsible for these efforts and the NIH survey team worked with all the groups contributing to the supplement on the COVID-19 pandemic in the current JCT issue. We contributed to this survey by focusing on questions specific to COVID-19 trials. The survey included as a supplemental table in the companion paper entitled “Re-engineering the Clinical Research Enterprise in Response to COVID-19: The Clinical Translational Science Award (CTSA) Experience and Proposed Playbook for Future Pandemics.”

The survey questions specific to this topic were developed by the authors to address key questions relevant to identifying COVID-19 research priorities and assessing implementation of COVID-19 research practices. These questions addressed such domains as establishment of COVID-19-specific research resources (e.g., funding, laboratory, clinical area, staffing), how decisions regarding COVID-19 research opportunities were managed and prioritized (e.g., which studies were implemented and which were not, recruitment of subjects for competing trials), and how the CTSAs were involved in decision-making or implementation. Our questions were reviewed and approved by a steering committee of the CTSA, and the survey was administered in REDCap. The survey was sent in late October 2020 to each CTSA to be completed within 2 weeks. We attempted to uncover important lessons learned from the individual centers and the collective experience using mixed methods. We identified some of the best practices and missteps that might guide the scientific community to manage the current and prepare for future pandemics. We conduced descriptive analyses, such as frequency count and chi-squared tests, and ran logistic regression to examine the role of the selected variables in establishing COVID-19-related research areas and diagnostic laboratories. Qualitative responses were grouped and analyzed by theme with emphasis on the role of CTSAs in prioritizing studies of COVID-19 and lessons learned. All analyses were conducted in STATA (Ver 14.2) [14].

## Results

A total of 64 CTSA hubs were invited to participate in the study. Of these, 60 (90% response rate) participated in the study. The response rate to the survey was very high, suggesting that the hubs did find this topic of great value. Our analysis indicates that early disruption of research (i.e., from January to March 2020) and institutional funding for COVID-19 were associated with the creation of a dedicated COVID-19 research area (Table 1). Institutions which provided emergency funding to their research community were 3.9 times more likely to create dedicated COVID-19 research areas (95% confidence interval [CI] = 1.1 to 13.4, *P* < 0.05). Likewise, early disruption from January to March 2020 was 8.1 times more likely to being associated with the creation of a dedicated COVID-19 research area (95% CI = 1.4 to 48.0, *P* < 0.05) as compared to the CTSAs not affected until after March 2020. Among all variables selected for the multivariate analyses, institutions with CTSA’s involvement in the institutional decision-making were 16.8 times more likely to create a COVID-19 diagnostic laboratory than those without CTSA’s involvement in the decision-making process. Among all funding sources, institutional funding was associated with the creation of a COVID-19 research area (Pearson χ^2^ ∼ 3.81; *P* ∼ 0.051). Federal funding was significantly associated with the creation of a new diagnostic laboratory (Pearson χ^2^ ∼5.7; *P* ∼ 0.017). When CTSAs were involved, 52.9% institutions hired new staff while no hires were reported in 47.1% when CTSAs were not directly involved in the COVID-19 response. Involvement of CTSA leaders in institutional decision-making was significantly associated with hiring new personnel for COVID-19-related studies (Pearson χ^2^ = 7.8088; *P* = 0.005). Fifty-two (86.7%) of the institutions reported establishment of a feasibility committee to prioritize review and performance of COVID-19-related studies. Active involvement of CTSA leaders in institutional decision-making was significantly associated with the formulation of such committees (Pearson χ^2^ ∼ 5.18; *P* ∼ 0.016).

**Table 1.** Role of the selected variables in establishing a COVID-19-related research area and a diagnostic laboratory. Odds ratios (95% confidence interval in parenthesis)

Variables	Creation of dedicated COVID-19 research area	Creation of COVID-19 diagnostic laboratory
Clinical and Translational Science Awards involvement in decision-making (1 = YES, 0 otherwise)	3.28	16.75***
	(0.52–20.59)	(2.17–129.39)
Funds provided by the institution (1 = YES, 0 otherwise)	3.88**	2.15
	(1.12–13.40)	(0.21–21.67)
Funds provided by the federal agencies (1 = YES, 0 otherwise)	1.37	4.74
	(0.33–5.66)	(0.56–40.51)
Time of COVID-19-related disruption (1 = Jan–March, 0 otherwise)	8.10**	1.19
	(1.37–47.98)	(0.08–18.20)
Constant	0.03***	0.22
	(0.00–0.34)	(0.01–6.28)
Observations	59	59

*** *P* ** , ** pns*P* ** pns*

Additional open-ended comments in the survey included the following: (1) prioritization of COVID-19-related research; (2) rapid startup; (3) whether previously approved human subjects research projects should continue; (4) facilitating information sharing and removing barriers; (5) securing funding; and (6) collaborating with other institutions and provide CTSA resources. Responses from 60 CTSAs focused on how they were involved in the decision-making process are presented in Table 2. Survey responses regarding sources of emergency funding received for COVID-19 research are summarized in Table 3.

**Table 2.** Survey responses from 60 Clinical and Translational Science Awards (CTSAs) with focus on how CTSA was involved in the decision-making process (survey free text)

Responses	Total, *n* = 48
Policy/guideline decision-making for research	17
Policy/guideline decision-making for institution/hospital and research	16
Prioritization of COVID-19-related research (approval of which studies to prioritize and engagement in rapid startup/execution of COVID-19 studies)	11
Decision-making process regarding which human subjects research projects could continue and which could not	5
Facilitated information sharing and removing barriers	4
Policy/guideline decision-making for hospital/university/institutional	3
Helped find funding	1
Engaged in collaborating with other institutions and local government	1
Deployed CTSA resources to assist with pandemic (i.e., Testing)	1

Total Responses* (some are counted more than once if response was multifaceted) = 48.

**Table 3.** Survey responses from 60 CTSAs with a focus on the source of emergency funding the institution received for COVID-19 research

Source	Number of responses
Benefactors/donors	16
Institutional	14
Pharmaceutical/industry	12
State	11
Award supplements (i.e., NCATS)	9
NIH	9
Federal	7
Foundation grants	7
CARES act	6
Local CTSA	5
BARDA	2
DOD	2
HRSA	2

COVID-19, The coronavirus disease 2019 AKA severe acute respiratory syndrome coronavirus 2 (SARS-CoV-2) disease; BARDA, Biomedical Advanced Research and Development Authority; CTSA, Clinical and Translational Science Award; NCATS, National Center for Advancing Translational Sciences; CARES act, Coronavirus Aid, Relief, and Economic Security Act; NIH, National Institute of Health; DOD, Department of Defense; HRSA, Health Resources and Services Administration.

A few CTSA sites (12%) had begun planning even before the first US case had been identified. Most hubs (86%) were engaged in the decision-making process at a leadership level for research. Some institutions (*n* = 26) reported having Biosafety Level (BSL)-3 facilities ready for use; but for others, such facilities had fallen into disuse. BSL-2 facilities could be rapidly enhanced to BSL-2+ facilities but were still insufficient for handling respiratory tract samples. Many institutions re-equipped their BSL-2+ facilities with the equipment to process and store samples for SARS-CoV-2 biorepository specimens but did not attempt to modify them to BSL-3 facilities. Discrepancies in requirements for similar procedures based on whether they were labeled research (NIH required) versus clinical (institution and CAP influenced) was a source of confusion. The CTSA hubs also participated in other key activities, such as feasibility assessments and drafting policies and standard operating procedures to conduct research.

Our data showed that human resources (HR) in many institutions allowed all interviewing and hiring to be performed with virtual technology. Some institutions used agencies and clinical research organizations to assist with staffing solutions, such as travel nurses, to fill time-sensitive research positions. In most institutions, COVID-19-related hires were fast-tracked with expedited on-boarding processes, including rapid hospital credentialing. Research staff from non-COVID-19 studies were often quickly redeployed to COVID-19 studies. Within divisions, staff was quickly shifted between studies, including redeployment from outpatient studies to inpatient trials, and then to outpatient vaccine studies. In some institutions, HR initiated a matching program between COVID-19 trial personnel needs and individuals who had been furloughed based on lack of work, allowing those at risk of termination to migrate to COVID-19 studies. Others pooled the coordinators, although sharing was not always easy due to competition for subjects among investigators conducting research that overlapped in inclusion criteria.

Despite efforts to rapidly hire staff for studies, some institutional Human Resources departments had difficulty navigating conflicting institutional policies regarding hiring and furloughs that differed for university-wide compared to hospital/medical research personnel. During the first 4 months of the pandemic, delays in dissemination of campus-wide policies regarding pay, leave of absence, and benefits slowed hiring. One institution suggested that during a pandemic, the School of Medicine should have been exempted from campus-level reviews to allow for timelier hiring for critical positions. Although some institutions awarded premium pay or compensatory time for evening and weekend work, many felt that compensation strategies failed to match the COVID-19 research needs.

In this study, 52 (86.7%) of the institutions reported establishment of a feasibility committee to review COVID-19-related studies to facilitate best practices. Involvement of the CTSA in the decision-making at a leadership level was significantly associated with the formulation of such committees. The scope of these committees also varied in that some reviewed therapeutic studies only (*n* = 24), whereas others reviewed all COVID-19-related studies (*n* = 12) that included databases and survey studies. These served as triage to limit duplication, optimize resources, and align with institutional priorities, patient availability, and staff capacity.

**Fig. 1.**
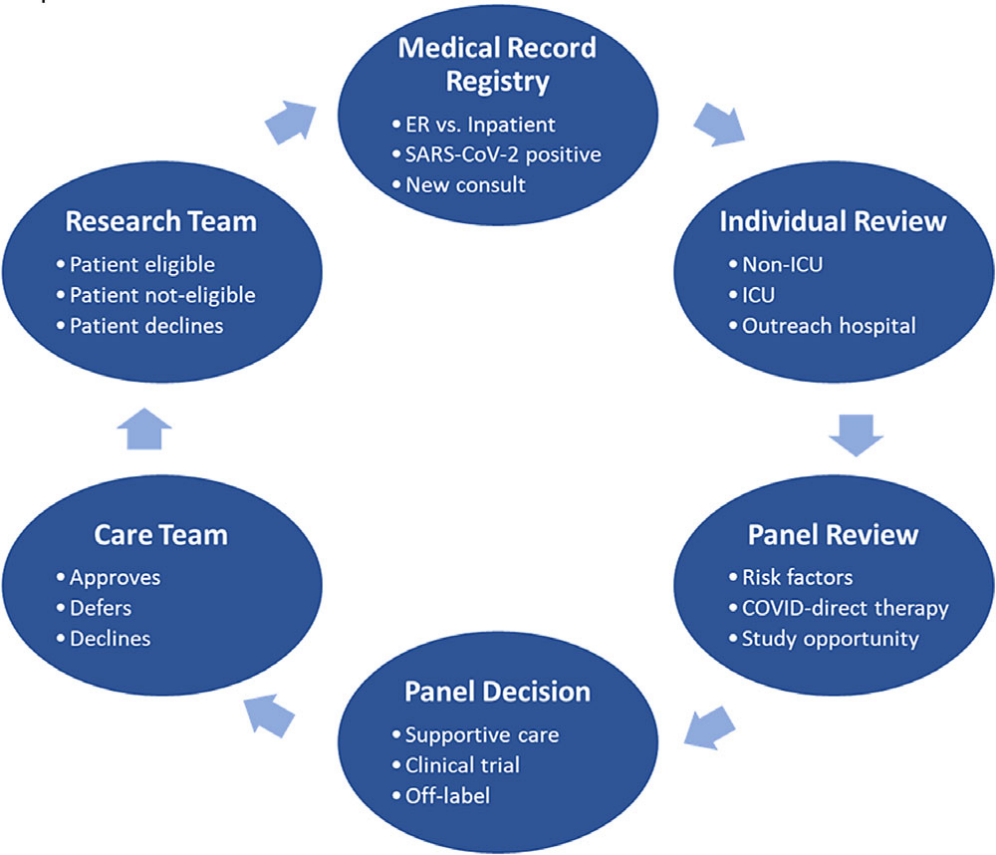
COVID-19 treatment review panel workflow (Mayo Clinic’s experience), In order to prioritize studies while maintaining equipoise among them, the establishment of ad hoc committee to review each new case in a multidisciplinary model that interacts with the care teams is a way to facilitate research as well as the best supportive care, adapted with time. ER, emergency room; ICU, intensive care unit.

For example, one institution declined a hydroxychloroquine trial in favor of trials using remdesivir, glucocorticoids, or lenzilumab. Some institutions implemented an expanded access program for convalescent plasma based on a national US registry, whereas others chose to participate in a randomized control trial on convalescent plasma. A typical example of such activity is shown in Fig. 1 used by one of the CTSAs.

When studies had overlapping inclusion/exclusion criteria, some institutions managed this by limiting the number of open trials with overlapping patient criteria. Other sites used a multidisciplinary team approach, including investigators and clinical providers, to offer patients the trial that seemed to best fit the patient’s preferences and treatment plan, while trying to avoid being overly influenced by one’s individual bias.

## Discussion

CTSA hubs, invited to participate in this survey, adopted approaches to address the COVID-19 pandemic directly and appear to have played a key role in the decision-making at the leadership level and the implementation of those decisions. Considerable heterogeneity existed in the readiness of hubs to adapt to the needs of COVID-19 research, as well as to the approaches each hub took to addressing hurdles related to pandemic research. Nonetheless, some common themes were expressed in these surveys as well as in recent literature that may offer guidance in how to prioritize pandemic research and demonstrate the lessons learned (Table 4).

**Table 4.** Lessons learned regarding prioritizing pandemic research using mixed methods

1.	Clinical and Translational Science Awards (CTSAs) played a major role in the current pandemic and should be empowered to do so in future pandemics with sufficient funding.
2.	Funding from benefactors/donors, institution, industry, National Institutes of Health/National Center for Advancing Translational Sciences and state were important sources for COVID-19 research. However, Clinical and Translational Science Institute funding made the greatest impact on infrastructure.
3.	Developing laboratory services, new diagnostics, biosafety level 2 to 3 laboratories, and biorepositories is essential in the preparedness of a pandemic.
4.	Most institutions can shut down their regular activity and engage their workforce in pandemic research instead.
5.	Flexible staff hiring and overtime are needed to facilitate enrollment into studies. Early engagement of human resources is essential.
6.	Formation of feasibility committees to process high study proposal volume and facilitate the assessment of the feasibility and scientific merit of potential studies.

*Personnel to facilitate COVID-19 research:* As COVID-19 spread across the country, provisions for pandemic staffing had to be addressed. COVID-19 studies were exempt from research shutdowns and policies that required limiting on-campus presence to maintain social distancing. Our data showed the involvement of CTSA in decision-making was significantly associated with hiring personnel in COVID-19-related studies. Institutions often implemented hiring freezes but still allowed new hires for patient-facing positions, grant-funded positions, and those funded by the National COVID-19 Cohort Collaborative.

*Assessment of study feasibility and prioritizing COVID-19 studies at the institutional level:* Given the rapid spread of the virus and early reports of severity of illness associated with infection, many potential studies were anticipated ranging from data analysis to interventional studies. Many institutions faced a swift closure of existing research (see article on Prioritizing Non-COVID-19 Research in this issue) and realized a keen interest among investigators to conduct COVID-19 research.

It is our view that recruitment of patients with COVID-19 who were eligible for a trial came with challenges. For outpatient clinical trials, often no clear relationship existed between the subject and the institution, and as such, an adequate method to contact these patients had to be developed. For inpatient trials, obtaining approval of the treating health care team became logistically challenging as the treating physician changed frequently in pandemic situations. Clinicians, patients, and families were not aware of the clinical trials; as such, communication was key, and an information sharing process needed to be developed so that all patients had the opportunity to participate if they desired. Some sites reported that it was highly recommended that clinicians advise patients of trial opportunities or at least be aware of patients’ interest in participating in clinical trials in general and guide them [15]. However, some clinical teams were not fully aware of clinical trial opportunities, and when under duress, research was not at the forefront of their treatment plans. With a new disease, a great need existed to assess the safety and efficacy of therapies through randomized controlled trials and to avoid the pitfalls of empirical treatment with untested therapeutics [16].

Given the abundance of clinical trials for COVID-19, there was potential for competing trials (i.e., subjects would be eligible for more than one trial) [17]. This raised an ethical dilemma, not new to research, but exacerbated by the pandemic. This problem was more complex because of the isolation of the patients, often secluded from family members under strict visitor restrictions [18]. Good research practice emphasizes the importance of equipoise, stating that one treatment is not *a priori* superior to the other and any potential benefit of a studied therapy might only benefit future patients [19]. Equipoise should also be respected when choosing between different potential trials for a given patient. Most institutions developed their own procedures to address this issue. The advice from one institution’s Clinical and Translational Research Ethics Consultation Service was that all study interventions, including controls, should have equipoise, meaning that no arm of the trial is believed to be more effective than any other. A positive balance of benefits against harms was ensured by Institutional Review Boards (IRB), and if the risks were similar among studies, there should be no reason to prefer one over another. To avoid bias and ensure equity and fairness among studies after ensuring that the patients met multiple study inclusion criteria and were interested in participating in a clinical trial, they were assigned to one of the eligible trials based on a predetermined randomization algorithm. There were several options for this process, such as the permuted block methodology [20, 21] or a consensus by adjudication through a committee. Care has to be given to avoid overwhelming critically ill patients and their caretakers with information about multiple trials competing for enrollment of patients.

Although IRBs are charged with protecting the rights of the persons between the arms of a trial, some COVID-19 committees did not believe that all open studies at a single institution could be equipoised. Changes were made to the regulatory process of IRB review in some institutions with a goal of reducing administrative barriers without compromising human subjects protections while facilitating urgent research (see the article on changes in IRB during the COVID-19 pandemic in this issue) [22]. The choice of selected studies within a given institution varied based on local context (scientific merit, local policies, and preferences). Based on the resources available and institutional priorities, multiple strategies were used to address competing trials. The ethics of setting priorities among competing studies with a therapeutic goal needed also to address other factors, such as the potential impact of a study on minorities or underserved populations or nontherapeutic studies (e.g., studies examining anxiety related to patient’s isolation from a caregiver). Such studies could still be considered high priority if they directly address patient needs occurring in special populations and could likely be done without negatively impacting recruitment for other studies. Lastly, as the number of COVID-19-related trials increased, the situation arose where the institutional committee were increasingly asked to make judgements regarding studies that might otherwise have been approved but for the likelihood that insufficient subject numbers were available.

*Creation of dedicated areas for clinical COVID-19 research:* Many institutions (50%) created centrally located spaces for clinical COVID-19 research (Table 1). These were mostly facilities normally used for clinical care that were repurposed for research. Initially, institutions did not permit patients with active COVID-19 in research spaces, but eventually made them available for research visits. However, hospitals varied as to the availability of these spaces for COVID-19 study visits. The lack of access to adequate outpatient space for such subjects was a considerable barrier to translational research. Moreover, many sites reported having to purchase equipment (e.g., freezers, centrifuges) specifically for COVID-19-related laboratory research. Our data suggested that institutions which provided funding were more likely to create a dedicated COVID-19 research area. Likewise, institutions that experienced early COVID-19 disruption (Jan–March 2020) were also more likely to create a dedicated COVID-19 research area.

*Diagnostic laboratories and biosafety adaptations to permit COVID-19 research*: In our multivariate analyses, institutions with CTSA involvement in decision-making were more likely to create a COVID-19 diagnostic laboratory than those with no CTSA involvement. This may have had a major impact on research and patient care; however, this came with added responsibility of compliance with biosafety and human subject protection following National Institutes of Health and institutional biosafety guidelines [23] during a pandemic. Measures taken to ensure the safety of staff and study subjects are addressed elsewhere in this issue [24]; however, there were some steps necessary for the conduct of COVID-19-specific research. Because the pandemic involved a novel respiratory virus, laboratory studies were restricted to BSL-2+/BSL-3 where available. Institutions with the existing and functioning BSL-3 facilities or previously used BSL-3 space were more likely to have the capacity to work with SARS-CoV-2 virus. Sites were more likely to rapidly open or expand BSL-3 lab space if they were already performing coronavirus research prior to the pandemic, had research programs that used other live viruses, or had developed this capacity previously (e.g., through Ebola-specific funding). No national list of BSL-3 facilities in the United States exists despite recommendations in 2014 to create a database of these facilities. Maintaining BSL-3 facilities is expensive; for example, training of individuals to work in a BSL-3 facility is estimated to cost $3000–7000 per worker. In addition, processes to document training for BSL-2+/BSL-3 are not streamlined, causing frustration among study teams and regulatory staff [25].

The pace of the pandemic caused an upending of our normal discipline with the development of therapeutics [15]. The stories of hydroxychloroquine [24, 26], convalescent plasma [27], and ivermectin [28] are good examples of where science and “hype” collide, jeopardizing the fundamental principle of evidence-based medicine at its core [29,30]. International adaptive platforms already in place such as REMAP-CAP [31] adjusted to incorporate COVID-19 infection. In the United States, the NIH initiated the Accelerating COVID-19 Therapeutic Interventions and Vaccines (ACTIV) public–private partnership to develop a coordinated research strategy for prioritizing and speeding development of the most promising treatments and vaccines [32]. CTSAs were invited to participate in that collaborative effort. Lessons learned from previous experiences such as Ebola preparedness [33, 34] helped the current pandemic but did not sufficiently lead to the kind of readiness required, to bring the pandemic under control.

*Institutions should be able to adapt to the needs of a highly contagious pandemic:* Given the great uncertainty and anxieties that staff may have about dealing with subject known or suspected to have a contagious disease, such as COVID-19, the institution must have the clinical and laboratory resources to evaluate these subjects and to handle and study biospecimens derived from them. For some, there will be laboratories and clinical spaces ready to accommodate, but for most others, there will need to be a repurposing of spaces to handle such investigation. These laboratories are needed in the immediate setting to inform diagnostic and biosafety practices, but also may provide for future research into understanding the sequelae of the condition.

*A pandemic is associated with human and emotional factors that need to be considered in future:* The most remarkable feature observed at the beginning of the pandemic was a combination of absolute enthusiasm to tackle this new and threatening infectious disease that propagated like a wildfire mixed with fear of the unknown, of being infected, or of bringing the infection home. At a time when most institutions shut down their regular activity, an unexpected workforce suddenly arose ready to engage in research. To channel this energy in such situations, research staff can be repurposed to investigate features of the pandemic or human resources must be flexible to meet hiring needs, whether through rapid new hires or creating overtime for existing staff.

*Enrolling patients into a variety of studies:* A multidisciplinary approach and close relationship between research investigators and clinicians are key to successful enrollment of patients into trials. In order to conduct clinical research at the bedside, investigators must obtain the consent from the patient (or representative) and assent of the treating clinician. COVID-19 offered several challenges to the conduct of research. Since some trials excluded some patients treated with therapies that had become standard of care (e.g., remdesivir, convalescent plasma, corticosteroids), there was little time to identify the subjects. Where competing trials existed, potential subjects needed to be educated on the opportunities available while maintaining equipoise in recruiting trials. Clinicians providing care for patients ill with COVID-19 may not have participated in the selection of trials, and therefore, may not agree with investigators on which, if any, trial might be of greatest interest or benefit to their patients. Programs generally engaged a multidisciplinary panel composed of infectious diseases and pulmonary specialists, intensivists, and *ad hoc* trialists to assist in defining treatment strategies and allocating patients into enrolling studies. In order to optimize patient’s allocation into trials, the mission of such a patient treatment panel should be to (1) guarantee that standard of care is applied to every patient as per institution’s policy to prevent heterogeneity between groups, (2) explore research opportunities using a hierarchy (randomized controlled trial against placebo, open label against standard of care, compassionate use), (3) adapt strategies based on daily literature reviewed through PubMed and national scientific societies, and (4) limit the number of trials to assure feasibility and equipoise with emphasis on coordination and communication between the multiple stakeholders.

## Conclusion

COVID-19 is not a short-term event and is likely not the last pandemic that we will encounter. What has become clear is that there was a need to have a state of readiness to meet the challenge. The CTSA network provides the ideal infrastructure to serve that purpose and should be fully integrated into university and hospital leadership decision-making. Institutions created various forms of a centralized review process to identify approaches to research that are best suited for their community. Although the optimal method and structure of an oversight committee may not be well established, a coordinated review process is required to make the best use of constraint resources. Constant review of evolving knowledge must be used to improve and refine processes.

As traditional methods to prevent the spread of infection (i.e., physical distancing and wearing of masks) were not been fully adopted, it appears that therapeutic interventions will be needed to tame the pandemic. Clinical trials designed and implemented with scientific rigor must be conducted without distraction from political background noise. We must have the ability to identify eligible subjects and offer them the opportunities available with the premise that we will adhere to Good Clinical Practice [35].

The future should focus on preparedness and on not repeating the errors. The CTSA network is well positioned to play a pivotal role in this goal, as it provides the infrastructure to rapidly mobilize in response to such emergencies and should be supported and utilized to protect the health of the nation.
